# Effect of acute ankle experimental pain on lower limb motor control assessed by the modified star excursion balance test

**DOI:** 10.3389/fspor.2023.1082240

**Published:** 2023-01-18

**Authors:** Michaël Bertrand-Charette, Jean-Sébastien Roy, Laurent J. Bouyer

**Affiliations:** ^1^ Center for Interdisciplinary Research in Rehabilitation and Social Integration, Québec, QC, Canada; ^2^ Department of Rehabilitation, Faculty of Medicine, Université Laval, Québec, QC, Canada

**Keywords:** motor control, pain, ankle, star excursion balance test, SEBT, ankle sprain

## Abstract

**Introduction:**

Following most musculoskeletal injuries, motor control is often altered. Acute pain has been identified as a potential contributing factor. However, there is little evidence of this interaction for acute pain following ankle sprains. As pain is generally present following this type of injury, it would be important to study the impact of acute pain on ankle motor control. To do so, a valid and reliable motor control test frequently used in clinical settings should be used. Therefore, the objective of this study was therefore to assess the effect of acute ankle pain on the modified Star Excursion Balance Test reach distance.

**Methods:**

Using a cross-sectional design, 48 healthy participants completed the modified Star Excursion Balance Test twice (mSEBT1 and mSEBT2). Following the first assessment, they were randomly assigned to one of three experimental groups: Control (no stimulation), Painless (non-nociceptive stimulation) and Painful (nociceptive stimulation). Electrodes were placed on the right lateral malleolus to deliver an electrical stimulation during the second assessment for the Painful and Painless groups. A generalized estimating equations model was used to compare the reach distance between the groups/conditions and assessments.

**Results:**

*Post-hoc* test results: anterior (7.06 ± 1.54%; *p* < 0.0001) and posteromedial (6.53 ± 1.66%; *p* < 0.001) directions showed a significant reach distance reduction when compared to baseline values only for the Painful group. Regarding the anterior direction, this reduction was larger than the minimal detectable change (5.87%).

**Conclusion:**

The presence of acute pain during the modified Star Excursion Balance Test can affect performance and thus might interfere with the participant's lower limb motor control. As none of the participants had actual musculoskeletal injury, this suggests that pain and not only musculoskeletal impairments could contribute to the acute alteration in motor control.

## 1. Introduction

Ankle sprains are frequent musculoskeletal (MSK) injuries (1–3). After an initial ankle sprain, approximately 33% of patients suffer from chronic ankle instability (4), reporting residual symptoms such as recurrent sprain, episodes of ankle joint “giving way,” pain, swelling, and decreased function (5). Chronic ankle instability can be perceived up to 3 years following the injury (4). Moreover, up to 78% of the individuals with ankle injuries are at risk of developing ankle osteoarthritis (6, 7). Therefore, adequate follow-up of people who sustained an ankle sprain is crucial to prevent chronicization and further damage at the ankle.

A wide variety of tests has been developed to assess individuals with ankle injuries. These tests can either assess somatosensation or motor control (8). Somatosensation tests imply the use and interpretation of sensitive information from sensory receptors such as muscle spindles, Golgi tendon organs, joint receptors and cutaneous receptors from skin over the joints (9). Somatosensation tests are useful following ankle sprain as this type of injury can further alter somatosensation (10). Motor control tests give information about the ability to regulate or direct the mechanisms essentials to movement (11), thus assessing performance during functional movement execution. A recent systematic review reported that the Star Excursion Balance Test (SEBT) is the most valid, reliable and responsive test to assess the lower limb motor control of individuals with a sprained ankle (8). Initially described with a participant standing on one leg and reaching as far as they can on a star-shaped form (12), this motor control test also has two short versions using a Y-shaped form showing similar psychometric properties, the modified Star Excursion Balance Test (mSEBT) (13) and the Y-Balance Test (14, 15). As the mSEBT is a reliable clinical tool to assess dynamic postural control, a recent review with practical guidelines suggested to use this short version instead of the 8-directions SEBT (13). All of these tests require little equipment and are easy-to-use in clinical settings (16).

Even if these tests seem promising regarding the assessment of sprained ankles, both of them have mainly been studied in healthy or chronic ankle instability populations (12, 14, 15, 17). Therefore, the impact of acute pain on reach distance and motor performance remains unknown. If the presence of pain interferes with ankle motor control, it could significantly reduce mSEBT reach distance and adversely affect score interpretation. Hodges and Tucker suggested that acute pain can cause changes in mechanical behaviour (18). These changes could increase muscle stiffness and induce a redistribution of load on joints or affect the direction of force vectors during movement. Such changes could therefore affect performance during the mSEBT.

Moreover, studying the effect of pain on motor control is of great interest as musculoskeletal pain is a major reason for consultation in primary care (19) and can be associated with reduced function (20). However, studying groups with musculoskeletal pain can be very challenging due to high rates of participants’ exclusion and to the difficulty to predict how painful a given task will be for each individual (21). Therefore, recruiting healthy participants could avoid these limitations, and allow assessment of the impact of pain on motor control under controlled conditions.

The main objective was to assess the effect of an acute electrical nociceptive pain at the ankle on reach distances during the mSEBT. To do so, participants were divided in three sub-groups (no pain, non-nociceptive electrical stimulation and nociceptive electrical stimulation) to complete two mSEBT and compare their reach distance between the two assessments (the first mSEBT is performed without stimulation for all groups). We hypothesized that if pain has a specific impact on motor control, only participants in the painful group would show a significant reduction for reach distances during the second mSEBT. Therefore, this hypothesis is related to the fact that pain, and not the electrical stimulation, could alter the mSEBT performance.

## 2. Material and methods

### 2.1. Participants

Sample size was calculated using G*Power 3.1.9.6 and based on a previous study to determine the optimal number of participants per group (22). A convenience sample of 48 participants was recruited from Université Laval student population. All included participants had to be (1) unaware of the research hypothesis, (2) be aged between 18 and 35 years old and (3) be free of any self-reported pain on the day of the experiment. Participants also had to (4) be free from any lower limb injury in the last 6 months, (5) be able to tolerate an experimental pain of 4/10 on the visual analog scale (VAS) for the duration of assessment and (6) be free of any movement limitation at the lower limb or any neurological impairment that could have affected task performance. Participants were excluded if they scored 71/80 or lower on the Lower Extremity Functional Scale (LEFS), a self-reported questionnaire used to assess lower limb function. The cut-off score of 71/80 was selected regarding its minimum detectable change (MDC) (23). All participants read and signed a consent form describing the experimental procedure and their involvement in the study. This protocol was approved by the local ethics review board (CIUSSS-CN, #2010-212). The experimental procedures were in accordance with the Declaration of Helsinki.

### 2.2. Modified star excursion balance test

**Figure 1:**
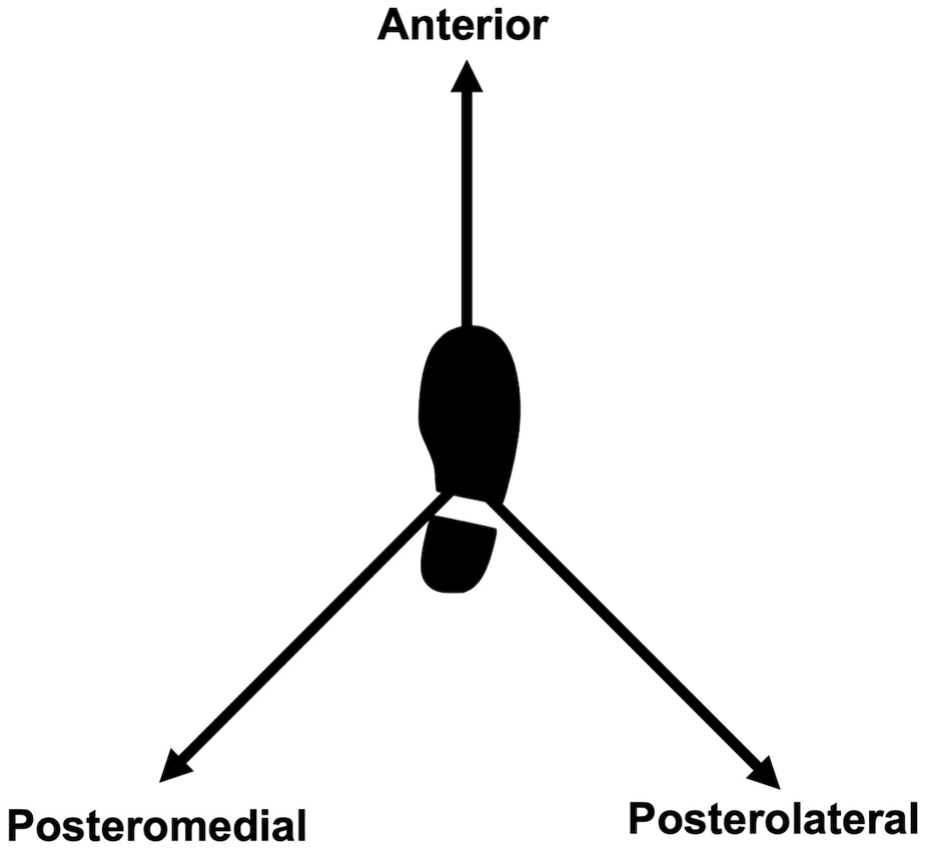
Representation of the *modified star excursion balance test* for the right weight bearing limb.

The modified Star Excursion Balance Test is a simplified version of the SEBT. Measuring tapes are placed on the floor in a Y-shaped form and participants have to stand on one foot (the one assessed) in the middle of the Y (Figure 1). They are asked to reach as far as they can on the measuring tape while maintaining balance, with their hands on their hips and the stance foot remaining flat on the ground. For a trial to be accepted, participants need to execute a controlled excursion on the Y-shaped form and lightly touch the ground with the tip of their foot as far as they can (24). If they lose balance or step on the measuring tape, they must repeat the trial.

### 2.3. General protocol

Participants were recruited for a laboratory session that lasted 45 min. Upon arrival, they read the general protocol and completed the LEFS and the Waterloo Footedness Questionnaire (WFQ) to assess foot preference (25). Then, a physiotherapist from the research team measured participants leg length and explained to the participants how to execute the mSEBT. They were asked to practice the mSEBT four times (26) while receiving verbal feedback from the physiotherapist to standardize the mSEBT execution.

Immediately following the practice period, participants had to complete the first mSEBT (mSEBT1). Since none of the participants had sprained ankles, all mSEBT were assessed while standing on the dominant limb based on the WFQ. All participants had to reach as far as they could on the measuring tapes. Reach distance was assessed a minimum of two times for each direction: Anterior (Ant), Posterolateral (PL) and Posteromedial (PM). Since maximum excursion distances values have usually achieved stability within the first 4 practice trials (26), participants were asked to complete each reach distance assessment twice. If the difference between two reach distance measurements was greater than the MDC (i.e., 6.46 cm for Ant, 9.28 cm for PL and 7.55 cm for PM) (8), a third and final attempt was made for this direction. The two closest values were kept for analysis.

After mSEBT1, participants had to remain seated for 15 min. During this break, they were randomly assigned to one of the three following groups: (1) *Control group*, in which participants completed a second mSEBT without any electrical stimulation; (2) *Painless stimulation group*, where participants completed a second mSEBT with a non-nociceptive electrical stimulation at the ankle; and (3) *Painful stimulation group*, in which participants would receive a nociceptive electrical stimulation at the ankle.

For the participants in Painless and Painful groups, during the 15-minutes break, stimulation electrodes were placed on the right lateral malleolus and at the distal end of the fibula of the dominant limb and the intensity of the electrical stimulation was calibrated; thereafter they were asked to complete mSEBT2 with the electrical stimulation. Participants in the Control group were asked to complete a second mSEBT (mSEBT2) following the break without any difference from mSEBT1.

### 2.4. Electrical stimulation

Two electrical stimulators (s-88, Grass Instruments, Quincy, MA, USA) were used to generate trains of 5 pulses at 300 Hz (pulse width 500µs) delivered through a Digitimer DS7A stimulator (Hertfordshire, United Kingdom) to an anode and a cathode placed two centimeters apart longitudinally over the right lateral malleolus and fibula. The electrodes placement was adjusted for each participant in a way that the pain would be local around the lateral malleolus (i.e., not causing radiating pain). Stimulation was triggered by a foot switch located under the dominant heel and was therefore present during each attempt. For the Painless group, increases in steps of 5 mA were used to individually adjust the stimulus intensity until the perception threshold (i.e., the lowest intensity at which each participant could feel the electrical stimulation) was reached. This 5 mA increment was delivered through a constant current unit in order to standardize the stimulus intensity increment, regardless of skin type or electrode quality. Final stimulus intensity was set at 1.2 times the threshold. For the Painful group, the same increases in steps of 5 mA were used until a pain level of 4/10 on the VAS was reached. For both groups, the intensity remained constant throughout the experiment. For more information regarding this experimental MSK-like pain protocol, see Bertrand-Charette et al. (27).

### 2.5. Recordings and data analysis

The physiotherapist assessing the mSEBT stood next to the participant during each attempt and noted the reach distance for each direction. Data were recorded for the raw score in centimeters and then normalized according to leg length, where the raw score was divided by the leg length and multiplied by 100 (28).

### 2.6. Statistics

**Figure 2:**
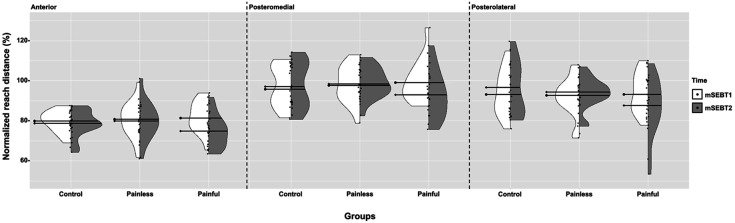
Violin plot representing mSEBT1 and mSEBT2 data. The distribution of mSEBT1 (white) and mSEBT2 (grey) data are presented for each group and for each direction. The thick black lines represent the median for each dataset (●- for mSEBT1 and ♦- for mSEBT2). Each dot represents the normalized reach distance for a participant.

First, to look at the overall distribution of data and guide the selection of statistical analysis, a violin plot (Figure 2) was built with packages ggplot2 (version 3.4.0, 2022-11-04), gridExtra (version 2.3, 2017-09-09) and the function GeomSplitViolin (https://github.com/iholzleitner/facefuns/blob/main/R/geom_split_violin.R) from the R statistical software (version 4.2.2, 2022-10-31). Then using IBM SPSS Statistics 29.0.0.0 (Armonk, NY), a repeated measures ANOVA designed for Gamma distributions (29) (GEE, generalized estimating equations) was used with Holm's sequential Bonferroni correction to compare normalized reach distances between two assessments, three directions and all groups. Some default parameters were changed as followed: DISTRIBUTION = GAMMA, LINK = LOG, and CORRTYPE = UNSTRUCTURED. The three independent variables were Group (between-subjects: Control, Painless and Painful), Time (within-subjects: mSEBT1 and mSEBT2) and Direction (within-subjects factor: Ant, PL and PM). Inherent pairwise comparisons to GEE model with Holm's Sequential Bonferroni were performed as *post-hoc* in the presence of significant GEE results. Significance level was set at 0.05.

## 3. Results

### 3.1. Sample size and participants’ characteristics

Following sample size calculation, a minimum of 15 participants per group was required to obtain statistical power of 0.95. Thus, forty-eight participants between the age of 19 and 34 years old were recruited for this experiment. Participants’ characteristics can be found in Table 1.

**Table 1:** Participants’ characteristics.

Characteristic	Control	Painless	Painful	*P*
*n*	16	16	16	n.s.
Age	27.5 ± 4.2	26.2 ± 4.4	26.8 ± 3.2	n.s.
Sex	8 M; 8F	8 M; 8F	8 M; 8F	n.s.
Footedness	15 R; 1 L	12 R; 4 L	16 R; 0 L	n.s.
Height (cm)	168.9 ± 11.5	174.3 ± 8.2	173.6 ± 8.9	n.s.
Leg length (cm)	91.9 ± 7.4	94.9 ± 5.6	95.4 ± 5.6	n.s.
Weight (kg)	69.1 ± 14.3	73.8 ± 10.8	71.2 ± 9.7	n.s.
LEFS score (/80)	78.3 ± 2.6	78.2 ± 2.4	78.8 ± 1.5	n.s.
Stimulation intensity (mA)	0	2.1 ± 1.1	9.8 ± 2.4	n.a.
VAS score during mSEBT2 (/10)	0	0	4	n.a.

F, female; L, left; M, male; R, right; n.a., not applicable; n.s., not significant VAS, Visual Analog Scale.

### 3.2. Stimulus intensity during the mSEBT2

Participants in the Painful and Painless groups received electrical stimulation during the second mSEBT. Painful group intensity was 9.8 ± 2.4 mA while Painless group intensity was 2.1 ± 1.1 mA.

### 3.3. Effect of pain on the normalized reach distances

Following the visual inspection of Figure 2, the GEE ANOVA was selected as it reported a far better goodness-of-fit statistic when using a Gamma distribution (log link; QICC = 39.39) compared to a normal distribution (identity link; QICC = 27104.50). Therefore, GEE analysis (see Table 2) was applied on the normalized reach distances, that is the raw score divided by the leg length and multiplied by 100 (28).

**Table 2:** *Post-hoc* test results for normalized reach distance.

Group	Direction	mSEBT1 [mean ± SD]	mSEBT2 [mean ± SD]	Sequential Bonferroni Sig.	95% Wald Confidence Interval for Difference		Mean difference (%) [mSEBT1–mSEBT2 ± SD]
					Lower	Upper	
Control	Ant	79.64 ± 5.30	78.59 ± 6.81	*p* > .05	−1.32	3.43	1.05 ± 0.96
	PL	93.98 ± 11.19	95.72 ± 13.20	*p* > .05	−4.65	1.18	−1.74 ± 1.03
	PM	97.24 ± 10.19	98.05 ± 11.66	*p* > .05	−2.53	0.91	−0.81 ± 0.68
Painless	Ant	80.50 ± 9.07	80.06 ± 9.54	*p* > .05	−0.81	1.69	0.44 ± 0.54
	PL	92.03 ± 10.06	93.72 ± 7.93	*p* > .05	−4.29	0.92	−1.69 ± 0.86
	PM	98.13 ± 9.29	98.97 ± 7.63	*p* > .05	−2.62	0.93	−0.84 ± 0.70
Painful	**Ant**	**81.92 ** **± ** **7.79**	**74.86 ** **± ** **8.27**	***p* = .00048**	**1** **.** **68**	**12** **.** **43**	**7.06 ± 1.54**
	PL	94.15 ± 10.07	86.77 ± 14.85	*p* > .05	−0.46	15.21	7.37 ± 2.30
	**PM**	**99.83 ** **± ** **10.47**	**93.30 ** **± ** **12.17**	***p* = .0075**	**0** **.** **80**	**12** **.** **26**	**6.53 ± 1.66**

Bold values represent statistically significant changes.

Group x Time interaction (*p* = .000011), and *post-hoc* tests, indicated that a significant difference between times (mSEBT1 and mSEBT2) happened strictly within the Painful group. No other statistically significant changes exist across groups when comparing within or between Control and Painless groups across mSEBT1 and mSEBT2 (see Supplementary File 1 for specific results). Therefore, all groups had similar reach distances at mSEBT1 (*p* > .05) and at mSEBT2 for Control and Painless (*p* > .05). On the contrary, regardless of direction, the Painful group showed a statistically significant change between mSEBT1 and mSEBT2 (7.03 ± 1.46% [2.76, 11.30], *p* = .00002).

As it was previously reported that Ant, PL and PM reach distances are affected differently by various factors (30), further *post-hoc* tests were performed for each direction to better understand this effect (Table 2). Moreover, effect sizes were examined as mean absolute differences with 95% confidence intervals reported between brackets. The *post-hoc* inherent pairwise comparisons reported no significant difference at all among the Control and Painless groups for all directions (*p* > .05) when comparing mSEBT1 and mSEBT2, while reach distance significantly decreased for the Painful group in the anterior (−7.06% [1.68, 12.43], *p* = .00048) and the posteromedial (−6.53% [0.80, 12.26], *p* = .0075) directions. The posterolateral distance showed a decrease of 7.34% which is consistent with the decrease seen in the two other directions although this difference is not statistically significant (*p* = .10).

## 4. Discussion

This study aimed to assess the effect of acute electrical nociceptive stimulation at the ankle on mSEBT reach distances. All groups performed two mSEBT separated by a 15-minutes break. However, only the Painful group showed significant reduction in reach distances during the second mSEBT. Our results suggest that acute pain could alter lower limb motor control.

### 4.1. Effect of pain on the reach distances

Participants in the Painful group showed a significant decrease in reach distance for two out of three mSEBT directions in the presence of an acute electrical nociceptive stimulation. This reduction ranged from 6.53% in PM to 7.06% in Ant for the normalized scores. These results are similar to previous studies comparing chronic ankle instability to a control group (17, 24, 31–33), where all participants with chronic ankle instability showed significant decrease in reach distances compared to controls. Only one study looked at the impact of acute ankle sprains on the reach distance (34). Similar to the chronic ankle instability studies (17, 24, 31–33), they noted a decrease in reach distance when comparing the sprained ankle group to the control group. However, there was no information regarding pain intensity from the participants in the acute ankle sprain groups making it hard to conclude on the impact of pain on the mSEBT reach distance. The presence of experimental acute pain in the present study caused a decrease in reach distance similar to what is seen with acute and chronic sprained ankles. This suggests that pain could negatively influence lower limb motor control even in the absence of mechanical limitation and that Ant and PM directions might be more affected by acute experimental pain than PL. However, it is important to note that the decrease seen in PL, even though not significant, is similar to the Ant and PM directions. Therefore, by recruiting more participants, PL could eventually show the same significant reach distance decrease. Moreover, a previous study showed that following ankle sprains, alteration in ankle motor control is not only the result of a peripheral deficit, but likely to be second to a reorganization of central motor commands, resulting in bilateral deficits during the SEBT (33). Therefore, pain and ligaments structural integrity both have the potential to interfere with motor control and general stability in sprained ankle participants.

#### 4.1.1. Clinical relevance

Another important finding in the present study is that the reach distance reduction caused by pain is greater than the minimal detectable change (MDC) of the SEBT for ANT direction. For example, the normalized score MDC for ANT has been reported to be 5.87% (35) while our results show a 7.06 ± 1.54% reduction in reach distance. The MDC is an estimate of the smallest change that falls outside the measurement error in the score, and it is based on the standard error of the mean (8, 36). Therefore, the mean 7.06 ± 1.54% shown in our results suggests that some participants had a reduction in reach distance with pain that was greater than the measurement error. It is also important to mention that the 95% confidence intervals were quite large, ranging from 1.68 to 12.43%. This supports the fact that pain is a personal experience (37) and that it might affect motor control differently, even across participants showing similar personal characteristics. These results, specific to the ANT direction, could suggest that this direction is the most affected by pain, in terms of motor control. As a matter of fact, a decreased performance in this direction has been shown to be related to an increased lower limb injury risk (38, 39). This direction is also highly affected by ankle dorsiflexion angle (40), a parameter shown to be reduced following chronic ankle instability and described as a predisposing factor for ankle injuries (41).

Finally, regarding the Painless and Control group, no significant changes were found in all three directions. This further support the hypothesis that pain and not just an electrical stimulation or distraction can alter motor control during a functional task. Moreover, none of these groups reach distance increased following the first mSEBT. This means that there was no learning effect throughout the study that could have affected the second execution of the test or the results.

### 4.2. Interaction between motor control and pain

Motor control is defined as the ability to regulate or direct the mechanisms essentials to movement (11). For proper regulation, timely integration of sensory information with movement planning and execution (i.e., sensorimotor integration) is necessary (8). The fact that spinothalamic projections to the motor cortex have been shown in humans (42) suggest that nociception should be considered both as a sensory input and also as a potential contributor to motor control. This contribution could either be beneficial or detrimental to performance during a functional task. In the current study, nociceptive inputs were detrimental to motor control during the modified Star Excursion Balance Test (a valid test used to assess motor control) by reducing reach distance in the Painful group. This interference of nociception on motor control is supported by neurophysiological studies [see Bank et al. (43) and Rohel et al. (44) for systematic reviews]. For example, M1 and S1 have been shown to exhibit decreased excitability in the presence of acute experimental pain (43, 45, 46). In addition, the Motor adaptation to pain model of Hodges and Tucker suggests that changes in mechanical behavior resulting from altered motor unit recruitment could be present around joints when pain is present (18). These changes could modify muscle stiffness and/or motor unit recruitment, here again affecting motor control. Finally, a recent study assessing proprioceptive acuity while walking demonstrated that pain can also interfere with sensorimotor integration during functional tasks (22). These studies, combined with the findings from the current study, demonstrate that pain interferes with sensorimotor integration and movement production, resulting in impaired motor control.

### 4.3. Strengths and limitations of the study

This study has some limitations. First, participants in all three groups were relatively young adult, which might limit the generalizability of the results. Another limitation is the absence of kinematic variables that could have added more detailed information on lower limb displacement during the mSEBT. Finally, the number of participants in each group was relatively small, resulting in large 95% confidence intervals for the mean absolute differences in reach distances.

This study also has several strengths. It is the first study to look at the effect of acute experimental pain on lower limb motor control (as assessed by the mSEBT). The presence of a group receiving non-nociceptive electrical stimulation allowed us to conclude that it is actually pain and not simply the electrical stimulation that specifically caused the modification in lower limb motor control. Finally, the use of an electrical nociceptive stimulation that caused a focused, acute and easily adjustable pain made it possible to control this pain intensity across participants in the Painful group.

## 5. Conclusion

Our results show that acute ankle experimental pain causes a reduction in mSEBT Ant and PM reach distances. This suggests that acute pain has the potential to interfere with lower limb motor control. Clinically, if the presence of pain interferes with ankle motor control, it could mean that the interpretation of the mSEBT reach distance should take into account the presence of pain, as it can significantly reduce the participant's ability to reach further. Further studies should include patients with acute painful ankle sprains to compare their results with the nociceptive electrical stimulation group to assess the effect of MSK pain on ankle motor control.

## Data Availability

The original contributions presented in the study are included in the article/**Supplementary Material**, further inquiries can be directed to the corresponding author.
